# Dietary of the Child in Health and Disease

**Published:** 1904-12

**Authors:** Herman B. Sheffield

**Affiliations:** Instructor in Diseases of Children at the New York Post-Graduate Medical School and Hospital


### DIETARY OF THE CHILD IN HEALTH AND DISEASE.

BY HERMAN B. SHEFFIELD, M.D.,

Instructor in Diseases of Children at the New York Post-Graduate Medical School and Hospital.

Healthy infants under nine months of age should be fed exclusively on milk. As salivary digestion is fully established at this age, a small quantity of carbohydrates in the form of a crust of stale bread or zwieback will certainly do no harm. When the child is over one year old an effort should be made, so to say, to teach the child to digest a few articles of food other than milk, in case an emergency arises when an exclusive milk diet may be contraindicated, as, for example, gastrointestinal disorders. Cereal gruel, soft-boiled egg, toasted bread, oatmeal crackers, strained chicken, mutton or beef soup, orange juice; and later, baked apple, baked potato with a little cream or sweet butter, will ordinarily be found suitable additions to a plain milk diet. Of course, the transition from an exclusive milk diet to a more or less mixed diet should be very slow and gradual, the effect of the change being watched from day to day and week to week, always bearing in mind that milk is the ideal food of the young child. Indeed, milk should be the chief constituent of the child's dietary until the sixth year, but beginning with the end of the second year the milk diet should from year to year gradually be displaced by the articles of food just mentioned, as well as by small quantities of chicken broth and mutton broth, scraped beef, rare steak, mutton chop, white fish, fresh vegetables, rice pudding, custard, cocoa, etc. All kinds of pastry, confectionery and fried food should be excluded as much and as long as possible from the dietary.

Children over one year and a half or two years of age very often discontinue drinking milk. This, I believe, is due chiefly to the fact that at about this age, generally upon the advice of the family physician, the child is forced to dispense with the bottle and nipple, its only companions for many months past. Why physicians have come to look upon bottles and nipples as a source of all evils to a child *over* one year, while readily sanctioning the use of bottles for children *under* that age, is to me a mystery! The mere fact that children continue to drink large quantities of milk until they are three or four years old, if given in a bottle; that taken through a nipple the milk enters the stomach very slowly and hence is better digested, and that finally, during sickness, milk as well as water (!) can best be administered by means of a bottle, justify me in the belief that the use of bottles for children of the ages mentioned should be encouraged rather than discarded, provided, of course, the bottles as well as the nipples are kept scrupulously clean—sterilized, if you please.

In feeding children during *acute illness,* nature's methou of induction of anorexia, while in that condition—obviously intended to prevent overfeeding while the digestive powers are greatly diminished—should be taken as a reliable guide. It is often surprising to see very delicate infants withstand a grave and tedious siege of sickness with hardly any nourishment at all. Like fish they seem to thrive on water, and this heavenly beverage should be given to them *ad libitum.* After a few days' illness, if the patient still refuses food, an attempt should be made to force it to take small quantities of liquid food, such as freely diluted milk, toast water, farinaceous water, albumin water, some artificial infant food of recognized value, peptonized or malted milk, etc. In breast-fed babies breast-milk, if need be, may be given by means of a spoon or dropper. Older children may be nourished also on strained cereal gruel, kumyss, matzoon, lactosomatose, eggnogg; chicken, mutton or beef soup; beef tea, beef jelly, water ices, ice-cream, fresh fruit juice, etc.

In delirium or stupor the child may be fed by lavage or per rectum. Rectal feeding is sometimes indispensable, for example, in diphtheritic paralysis, severe attacks of convulsions, etc., when feeding by the mouth may give rise to aspiration pneumonia, but it should be employed only as a last resort, since in young children it is very apt to produce irritation of the rectum. Furthermore, infants rarely retain nutrient enemas for any length of time. Peptonized milk, or, in older children, milk and egg or somatose, in quantities of from one to two ounces, may be injected into the rectum at intervals of from three to four hours, preceded by a high rectal irrigation. To check excessive peristalsis, a minute dose of deodorized tincture of opium may be added. The injection should be given at the temperature of the body, thrown in very slowly and as high up into the intestine as possible by means of a small-sized rectal tube and funnel, and retained by pressing the buttocks for at least half an hour afterwards.—*The Post Graduate.*

